# Gas6 induces inflammation and reduces plaque burden but worsens behavior in a sex-dependent manner in the APP/PS1 model of Alzheimer’s disease

**DOI:** 10.1186/s12974-022-02397-y

**Published:** 2022-02-07

**Authors:** Laura D. Owlett, Berke Karaahmet, Linh Le, Elizabeth K. Belcher, Dawling Dionisio-Santos, John A. Olschowka, Michael R. Elliott, M. Kerry O’Banion

**Affiliations:** 1 Del Monte Institute for Neuroscience, Department of Neuroscience, University of Rochester, Rochester, NY USA; 2 Department of Microbiology, Immunology, and Cancer Biology, University of Virginia, Charlottesville, VA USA

**Keywords:** Alzheimer’s disease, Microglia, Neuroinflammation, TAM receptors

## Abstract

**Background:**

Alzheimer’s disease is the leading cause of dementia worldwide. TAM receptor tyrosine kinases (Tyro3, Axl, MerTK) are known for their role in engagement of phagocytosis and modulation of inflammation, and recent evidence suggests a complex relationship between Axl, Mer, and microglial phagocytosis of amyloid plaques in AD. Gas6, the primary CNS TAM ligand, reduces neuroinflammation and improves outcomes in murine models of CNS disease. Therefore, we hypothesized that AAV-mediated overexpression of Gas6 would alleviate plaque pathology, reduce neuroinflammation, and improve behavior in the APP/PS1 model of Alzheimer’s disease.

**Methods:**

Adeno-associated viral vectors were used to overexpress Gas6 in the APP/PS1 model of Alzheimer’s disease. Nine-month-old male and female APP/PS1 and nontransgenic littermates received bilateral stereotactic hippocampal injections of AAV-Gas6 or AAV-control, which expresses a non-functional Gas6 protein. One month after injections, mice underwent a battery of behavioral tasks to assess cognitive function and brains were processed for immunohistochemical and transcriptional analyses.

**Results:**

Gas6 overexpression reduced plaque burden in male APP/PS1 mice. However, contrary to our hypothesis, Gas6 increased pro-inflammatory microglial gene expression and worsened contextual fear conditioning compared to control-treated mice. Gas6 overexpression appeared to have no effect on phagocytic mechanisms in vitro or in vivo as measured by CD68 immunohistochemistry, microglial methoxy-04 uptake, and primary microglial uptake of fluorescent fibrillar amyloid beta.

**Conclusion:**

Our data describes a triad of worsened behavior, reduced plaque number, and an increase in proinflammatory signaling in a sex-specific manner. While Gas6 has historically induced anti-inflammatory signatures in the peripheral nervous system, our data suggest an alternative, proinflammatory role in the context of Alzheimer’s disease pathology.

**Supplementary Information:**

The online version contains supplementary material available at 10.1186/s12974-022-02397-y.

## Background

Late-onset Alzheimer’s disease (AD) continues to pose a significant challenge to patients, affecting more than 5.8 million Americans and contributing over $300 billion annually to healthcare costs (2020 Alzheimer’s Disease Facts and Figures, Alzheimer's Association). Accumulation of amyloid species into plaques is one of the histopathological hallmarks of AD and is required for post-mortem diagnosis [1]. Human and mouse studies demonstrate that microglia, the immune cells of the brain, physically surround amyloid plaques and have been shown to promote plaque clearance through phagocytosis [2]. Furthermore, genome-wide association studies implicate genes critical to microglial phagocytosis in conferring increased risk for AD [3]. Despite this putatively beneficial role of microglia in reducing AD pathology, microglial production of inflammatory cytokines contributes to synapse loss and neurodegeneration [4, 5]. Thus, investigation of microglial targets to promote plaque clearance and reduce production of damaging cytokines may provide new avenues for drug development.

The TAM (Tyro3, Axl, Mertk) family of receptor tyrosine kinases has been well studied for their roles in cancer biology and autoimmune disease, and a number of studies implicate a role for these receptors in the pathogenesis of Alzheimer’s disease [6, 7]. Activation of Axl by its ligands induces canonical RTK signaling, which promotes proliferation and cell survival, actin mobilization and induction of phagocytosis, and reduction of proinflammatory cytokine expression [6, 8, 9]. In humans, plasma Axl levels correlate with increased [11C]-PiB (fibrillar amyloid beta) PET measurements, and Axl expression changes are noted in aged microglia, plaque-associated microglia from patients with early-onset AD, and other dementia variants such as hippocampal sclerosis [10–12].

In vivo studies suggest that both jujuboside A, an herbal medicine, and bexarotene, an antineoplastic agent, alleviate amyloid pathology through Axl-dependent mechanisms [13, 14]. More recent evaluation of APP/PS1 Axl^−/−^/Mertk^−/−^ mice has revealed the complexity of Axl signaling in the context of AD. Microglial processes from APP/PS1 Axl^−/−^/Mertk^−/−^ mice lack orientation to amyloid plaques, which indicates reduced phagocytic capacity of Axl^−/−^/Mertk^−/−^ microglia [15]. Surprisingly, however, APP/PS1 Axl^−/−^/Mertk^−/−^ mice have fewer dense-core plaques, which are now thought to be formed in part from microglial deposition of amyloid beta via exocytosis [15, 16].

Several lines of evidence suggest that altering levels of Gas6, the primary ligand for Axl and MerTK in the brain, may be promising for improving AD pathology. Gas6 is produced and secreted from neurons in the CNS and activates downstream pathways that engage phagocytic machinery and suppress inflammation [8, 17]. Treatment of cortical neurons with Gas6 causes a reduction in Aβ-induced apoptosis [18]. Administration of Gas6 has also shown benefits in reducing neuroinflammation and improving behavior in rodent models of stroke, acute cerebral hemorrhage, and multiple sclerosis [19–21]. Interestingly, Gas6 binds amyloid plaques in murine models and its production is tightly regulated by Axl expression [15]. However, no study to our knowledge has investigated the direct effect of Gas6 overexpression on behavior and pathology in the context of AD. Thus, we hypothesized that AAV-mediated overexpression of Gas6 in the hippocampus would alleviate plaque pathology, reduce neuroinflammation, and improve behavior in the APP/PS1 murine model of Alzheimer’s disease.

We induced bilateral hippocampal overexpression of Gas6 in the APP/PS1 model of Alzheimer’s disease with stereotactic injections of an adeno-associated virus containing Gas6. Although Gas6 overexpression reduced plaque burden in male APP/PS1 mice, contrary to our hypothesis, it increased pro-inflammatory microglial gene expression and worsened contextual fear conditioning compared to control-treated mice.

## Methods

### Animals

All animal procedures were reviewed and approved by the University Committee on Animal Resources of the University of Rochester Medical Center for compliance with federal regulations prior to the initiation of the study. Tg(APPswe,PSEN1dE9)85Dbo male mice were obtained from Jackson Laboratories (43832-JAX, C57Bl/6J background) and bred to female C57Bl/6J mice to produce a total of 38 APP positive mice and 29 nontransgenic mice. All mice were co-housed in cages of up to five mice and provided food and water ad libitum. For viral vector validation, 16 12-week-old C57Bl/6J male mice were used.

### Adeno-associated viral constructs

Gas6(Myc-DDK-tagged)-mouse growth arrest specific 6 (Gas6) cDNA was purchased from OriGene (Rockville, MD) and used to construct an AAV-packaging vector using In-Fusion HD Cloning Kit (Clontech Laboratories). The cloning vector was packaged into AAV-1 under the *SYN1* promoter at the National Institute of Drug Abuse at the NIH by Brandon Harvey, as described [22]. A deletion mutant lacking the Gas6 Gla and EGF domains, as described in [23], was created and processed as above.

### Viral vector validation study

Sixteen 12-week male C57Bl/6J mice received either 1.5 μL of 10E10 vg/mL or 10E12 vg/mL via stereotactic hippocampal injection as described below. Mice were sacrificed 4 weeks later. Half brains were processed for immunohistochemistry and the other half was processed for western blot, as described below.

### Stereotactic injections

The following protocol was used for all viral vector injections: mice were injected with slow-release buprenorphine (0.5 mg/kg, i.p.) and anesthetized with 1.75% isoflurane in 30% oxygen and 70% nitrogen. The head was secured in a Kopf stereotactic apparatus using ear bars and a head holder. Ophthalmic ointment was applied to prevent eye dryness. Hair was removed on top of the skull and the scalp was disinfected with betadine and ethanol prior to incision with a scalpel. Two 0.5 mm burr holes were drilled in the skull at 1.8 mm caudal and ± 1.8 mm lateral relative to bregma. A 33 GA needle was lowered over the course of 2 min to a depth of 1.5 mm. A Micro-1 microsyringe pump controller (World Precision Instruments, Sarasota, FL) was used to inject 1.5 μl of AAV-Gas6 or AAV-control (1E12 vg/mL, or 1E10 vg/mL for viral vector validation study) over ten min, followed by a five min rest period. After the injection, the needle was raised over the course of 2 min and the protocol was repeated on the opposite side of the brain. After injections were complete, the burr holes were sealed with Ethicon bone wax and the incision was closed with tissue adhesive. Mice recovered in a heated area before being placed in their home cage. Aliquots of AAV-Gas6 or AAV-control were randomized into microfuge tubes by another laboratory member so that the investigator was blinded to each mouse's treatment until the end of data collection. The order of injections was also randomized to control for time of day effects. Mice were monitored for total recovery for 3 days. All 77 mice were injected over the course of 1 week at 9 months of age. For the viral vector validation study, 16 12-week-old male mice were injected over the course of 2 days.

### Behavioral assays

Two weeks before all behavioral assays, mice were transferred to a reverse light dark cycle room so that behavioral assays could be run during the awake cycle. Four APP/PS1 male mice were eliminated from novel object and fear conditioning behavioral analyses (one in the control-treated group and three in the Gas6-treated group) due to observation of seizures during behavioral testing. The incidence of observed seizure behavior was 14% in the control-treated male APP group (1/7) and 23% in the Gas6-treated male APP group (3/13); no seizures were observed in the wild type control mice.

### Open field

To assess activity level and anxiety, an open field test was performed. Mice were allowed to explore freely in a 31 × 31 cm box for five min. AnyMaze software (Stoelting, Wood Dale, IL) was used to track mice and the periphery of box was defined by 5 cm from the edge of the box. Distance, mean speed, time spent freezing, and time in periphery was automatically determined using AnyMaze using the center of the mouse’s body. Boxes were cleaned thoroughly with ethanol between animals and male animals were run before females.

### Novel object recognition

During the habituation phase, mice were allowed to explore a 31 × 31 cm box for 5 min containing two identical objects spaced ~ 15 cm apart. All objects used were ceramic doorknobs of 5–6 cm in height and ~ 3 cm in width. Objects and chambers were washed with 70% ethanol before each trial. Two hours after the habituation phase, each mouse was returned to the experimental cage containing the object to which it was previously exposed (familiar object) as well as a novel object. Placement of the novel object was randomized for each mouse. Mice were allowed to explore familiar and novel objects during a 5 min test that was videotaped for subsequent analysis using the AnyMaze software. Scoring of NOR performance was based on time spent exploring both familiar and novel objects. The behavior of the mouse was considered exploratory when the animal’s head faced the object with the neck extended. Simple proximity, passing-by, or standing on the object did not count as exploration. Mice that spent less than 20 s exploring both objects during either the training or testing phase were not included in the analysis (1 APP/PS1 AAV-control male mouse, 3 APP/PS1 AAV-Gas6 male mice, 1 wild type AAV-Gas6 male mouse, 1 APP/PS1 AAV-control female mouse, 1 wild type AAV-control female mouse, and 1 wild type AAV-Gas6 female mouse). Discrimination index was defined as *(Time with NO* *−* *Time with FO)/(Time with NO* + *Time with FO)*.

### Contextual fear conditioning (CFC)

Mice underwent cued and contextual fear conditioning, as previously described [24]. Briefly, on conditioning day, mice were allowed to explore the conditioned context, which consisted of a Plexiglas chamber and metal floor grid (model H10-11M; Coulbourn Instruments, Whitehall, PA, USA). After 3 min, 15 s of white noise (80 dB) was presented co-terminating with a 2 s, 0.75 mA foot shock. This noise-shock pairing was repeated twice for a total of 3 shocks with a 30 s interval between shocks. Twenty-four hours later, mice were re-exposed to the conditioning chamber for 5 min and freezing responses were measured using FreezeView Software (Coulbourn Instruments) to test contextual long-term memory. Four hours later, mice were placed in a novel context consisting of a 15 cm open-topped plastic cylinder with bedding on the floor for 3 min followed by re-exposure to the white noise for 3 min, to test hippocampal-independent memory. All data were video recorded using FreezeFrame Video-Based Conditioned Fear System and analyzed by FreezeView Software (Coulbourn Instruments). CFC was performed in all male mice before female mice.

### Methoxy-04 injection and flow cytometry

After behavioral testing, and 24 h before sacrifice, animals were intraperitoneally injected with Methoxy-04 (Tocris Bioscience) at 4.35 mg/kg. On the day of sacrifice, mice were deeply anesthetized with xylazine (10 mg/kg) and ketamine (100 mg/kg) and perfused intracardially with 0.15 M phosphate buffered saline containing 0.5% sodium nitrite (weight/volume) and 2 IU heparin/mL. After perfusion, brains were collected and bisected along the longitudinal fissure. For all animals, one half brain was submerged in 4% paraformaldehyde, pH 7.2 in PBS at 4 °C for 24 h, and processed for immunohistochemistry as described below. For half of the animals, hippocampi from the other half of the brain were dissected and processed for ELISA as described below. For the other half of the animals, each hippocampus from the other half brain was processed for flow cytometry and RNAseq as follows: hippocampi were dissected and Dounce homogenized in 3 mL FACS buffer (PBS + 0.5% BSA). Brain homogenates were filtered through a 70 µm filter, rinsed with FACS buffer, and centrifuged at 400×*g* for 5 min at 4 °C. Pellets were resuspended in 40% percoll (GE Healthcare) and centrifuged at 500×*g* for 30 min without brake. Supernatants were removed and pellets were resuspended in 100 μL FACS buffer containing 1:100 Fc block (2.4T2, BD) and transferred to a 96 well plate. The following antibodies were added: CD45 (30-F11, APC/Cy7 1:400, Biolegend), CD11b (M1/70 Biolegend) and incubated for 30 min at 4 °C. Cells were washed once and stained with the 7AAD viability dye (ThermoFisher). They were then sorted on a BD FACSAria II (BD Biosciences) and gated on CD45^lo^/CD11b^+^ following exclusion of debris, doublets and dead cells. Cells were sorted into Buffer RLT Plus (Qiagen) + 10 μL/mL beta-mercaptoethanol and stored at − 80 °C until processing for RNAseq. Because two separate cytometers were used to collect data, a resolution metric (R_d_) was calculated as (Median_Pop2_-Median_Pop1_)/(rSD_Pop2_ + rSD_Pop1_), with MFI for methoxy-negative samples as Pop1 and MFI of methoxy-positive samples as Pop2.

### Tissue processing and immunohistochemistry

For immunohistochemistry, fixed half brains were equilibrated in 30% sucrose in PBS overnight, frozen in cold isopentane, and stored at − 80 °C until sectioning into 30 μm sections on a − 25 °C freezing stage microtome. Free-floating sections were stored in a cryoprotectant solution until assayed. For staining, sections were washed in PBS and blocked with normal donkey or goat serum for 1 h at room temperature. Sections were then incubated in primary antibodies (goat anti-Gas6 1:500, R&D Systems AF986-SP; rabbit anti-Iba-1 1:2000, Wako NC9288364; or rat anti-CD68, 1:500, Abcam ab53444), for 48 h at 4 °C, after which they were washed and incubated in secondary antibodies (Invitrogen Alexa fluors 488 or 594, 1:2000) for 2 h at room temperature.

### Image acquisition and analysis

Slides were imaged on an Axioplan Iii (Carl Zeiss, Oberkochen, Germany) microscope. Slidebook software (Intelligent Imaging Innovations, Denver, CO) was utilized for image acquisition, in which × 10 or × 40 images were taken of 3–4 sections with 5–6 plaques per mouse. Slides were labeled with blinded animal numbers. Images were analyzed using ImageJ software (National Institutes of Health, Bethesda, MD). For plaque % area and particle analysis, ROIs were drawn using the bottom leaflet of the dentate gyrus and upper CA1 field as anatomical guidelines. Images were then thresholded using ImageJ’s “Otsu” algorithm and % area and particle statistics were measured. For CD68 and Iba-1 quantification, ROIs were drawn around each plaque and enlarged such that each ROI encompassed the 15 μm surrounding each plaque. Images were thresholded using the “Otsu” algorithm. % area and integrated density were measured.

### Western blot

For the viral vector validation study, dissected hippocampi were flash frozen in ice cold isopentane and stored at − 80 °C. Frozen hippocampi were weighed and homogenized in T-PER (Thermo Scientific, 50 mg/mL) for 30 s, vortexed, and sonicated for 30 s. Protein concentration was determined using BCA assay (Thermo Scientific). Protein was then diluted in 2 × sample buffer (125 mM Tris–HCl; 4% SDS; 20% glycerol) at a concentration of 1 μg/μL. Samples were briefly vortexed, boiled for 10 min, and 15 μL was electrophoresed on a Tris–HCL polyacrylamide gel. Gels were transferred to a 0.4 μm PVDF membrane at room temperature overnight. Membranes were washed and blocked in 5% BSA/TBS-T for 1 h at room temperature. After washing in TBS-T, membranes were incubated in pAxl (Invitrogen PA5-64862) at 1:100 overnight at 4 °C. Membranes were then washed and incubated in peroxidase-linked secondary antibodies (Invitrogen) for 1 h at room temperature. Blots were treated with ECL substrate (Supersignal West Dura Kit, Thermo Scientific) and bands were visualized using an Azure c600 Gel Imaging System (Azure Biosystems, Dublin, CA). Blots were stripped for 10 min in Strip Reblot Plus Strong Solution (Miltenyi). Blots were then re-probed with mouse anti-Axl (R&D) at 1:100 and protocol above was repeated.

### ELISA

Hippocampal lysates from Gas6 and control-treated APP/PS1 mice were centrifuged at 100,000 g for 1 h at 4 °C to separate soluble aggregations of Aβ (monomers and oligomers) from large, insoluble Aβ fibrils. Supernatants were collected as the soluble fragments. CXCL13 and CCL2 chemokine levels were measured utilizing respective mouse ELISA kits (R&D MCX130 and MJE00B). Soluble samples were diluted 1:1 in kit buffer. Plates were read with Microplate Absorbance Reader (Bio-Rad). Linear regression models were used for CXCL13 & CCL2.

### RNAseq

Total RNA was isolated using the RNeasy Plus Micro Kit (Qiagen, Valencia, CA). RNA concentration was determined with the NanoDrop 1000 spectrophotometer (NanoDrop, Wilmington, DE) and RNA quality assessed with the Agilent Bioanalyzer 2100 (Agilent, Santa Clara, CA). One ng of total RNA was pre-amplified with the SMARTer Ultra Low Input kit v4 (Clontech, Mountain View, CA) per manufacturer’s recommendations. The quantity and quality of the subsequent cDNA was determined using the Qubit Fluorometer (Life Technologies, Carlsbad, CA) and the Agilent Bioanalyzer 2100 (Agilent, Santa Clara, CA). 150 pg of cDNA was used to generate Illumina compatible sequencing libraries with the NexteraXT library preparation kit (Illumina, San Diego, CA) per manufacturer’s protocols. The amplified libraries were hybridized to the Illumina flow cell and sequenced using the NovaSeq6000 sequencer (Illumina, San Diego, CA). Single end reads of 100 nt were generated for each sample. Two to six biological replicates were sequenced for each group.

### Bioinformatic analyses

The RNAseq bam file was mapped to mm10 reference genome obtained from Ensemble with STAR [25]. featureCounts was used to assign reads to genomic features [26]. DESeq2 was used to perform differential expression analysis [27]. Groups compared were AAV-Gas6-treated APP and AAV-control-treated APP. Genes were considered differentially expressed if *p*_adj_ < 0.05. Differentially expressed genes were separated based on the direction of their fold change and gene ontology analyses on the resulting lists of genes were conducted with clusterProfiler package [28].

### Primary microglial isolation

C57BL/6J Axl^+/-^ and Axl^−/−^ pups (Jackson labs: Axl^tm1Grl^/J, generously donated from the Calvi Lab at the University of Rochester) ages P0–P2 were sprayed with 70% ethanol and decapitated. Brains and meninges were removed. Tissue was washed with ice cold HBSS three times. 1 ml of 0.25% trypsin + 50 µL DNase was added and incubated at room temperature for 7 min. Tissue was again washed twice with HBSS. Tissue was dissociated using a P1000 pipettor by pipetting up and down until tissue was fully dissociated, then was filtered through a 70 µm nylon cell strainer into a new tube. DMEM + 10% FBS + 1% pen/strep was added and samples were spun at 300×*g* for 10 min. Samples were then transferred to vented T-75 flasks. Media was changed after 24 h after which cells were left to incubate for 2 weeks. At this time, microglia were isolated from astrocytes using a mild trypsinization protocol: cells were rinsed with DMEM without FBS, and incubated in 1 × trypsin/EDTA (Invitrogen 25200-056) diluted 1:3 with DMEM without FBS and incubated at 37 °C until the astrocytic layer was fully removed from the microglial layer (20–40 min) [29]. Microglia were then removed with undiluted 0.25% trypsin, collected, spun at 300 g for 10 min, and plated into cell-culture treated 96 well plates with serum-free media for 24 h before treatment. Cells were then treated with beta-amyloid (1–42) HiLyte Fluor 488 (AnaSpec) with or without 50 nM rmGas6 (R&D Systems) for 15 min, 30 min, 60 min, or 120 min. Cells were then washed and collected for flow cytometry, where they were analyzed using an LSR II (Becton Dickinson) in the University of Rochester Medical Center Flow Cytometry Core. Data was acquired using FCS Express 7 (De Novo Software, Pasadena, CA).

### Statistics and data analysis

All statistical comparisons, except for those pertinent to RNAseq data, were performed with Prism (GraphPad Software, version 8, San Diego, CA) using unpaired Student’s *t* tests, one-way ANOVAs, and two-way ANOVAs, as required based number of experimental vs control conditions. The Shapiro–Wilk test was used to determine normality of the data. Based on results of normality test, Student’s *t*-test or Mann Whitney test were employed when two group means were compared. Tukey’s correction for multiple comparisons was conducted where appropriate. All results are expressed as mean ± SEM. A *p*-value of < 0.05 was considered significant in all experiments.

## Results

### Effect of Gas6 overexpression on open field task and novel object recognition tasks

**Fig. 1:**
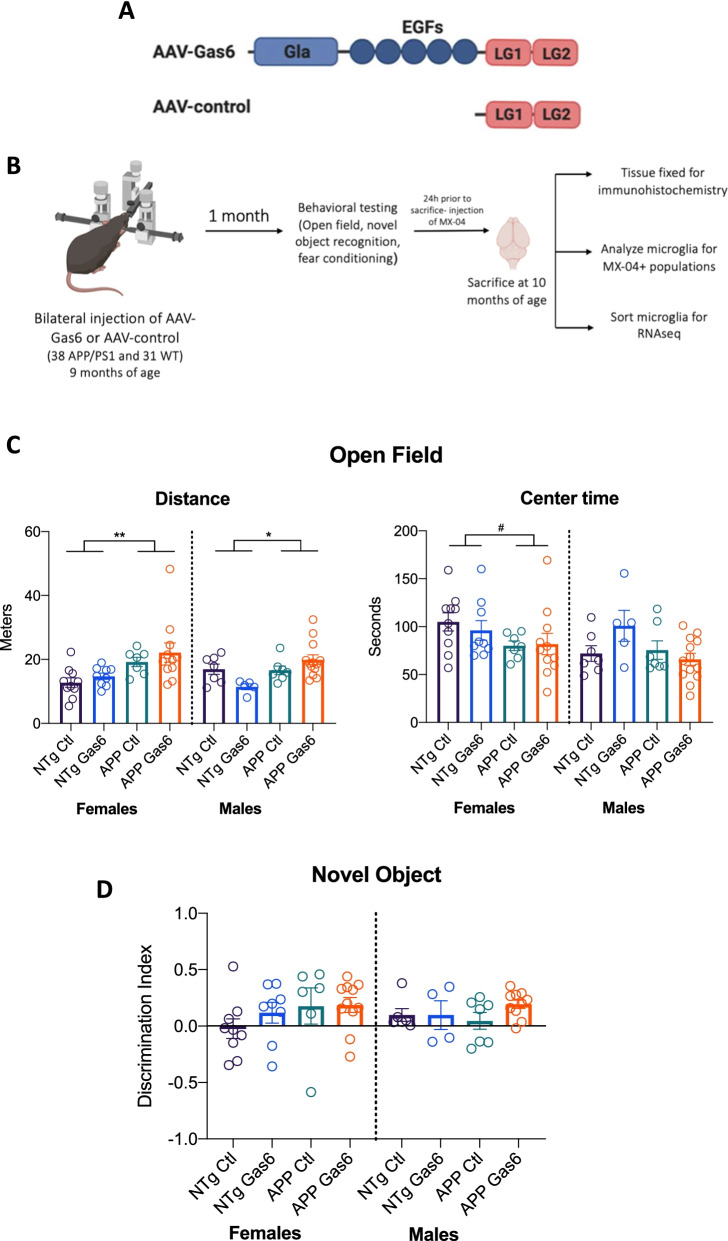
Effect of Gas6 overexpression on open field task and novel object recognition task. **A** Schematic of AAV-Gas6 construct containing full length Gas6 protein and AAV-control which contains an attenuated Gas6 sequence without the Gla and EGF domains. **B** Schematic of experimental design: APP/PS1 male and female mice received bilateral hippocampal injections of either AAV-Gas6 or AAV-control at 9 months of age. Behavioral tests were run 1 month later, and all mice were sacrificed at 10 months of age. **C** Mice were allowed to freely explore a 31 × 31 cm box for 5 min, during which distance travelled and time spent in the center of the box were quantified. *N* = 7–10 mice per group. Error bars represent mean ± SEM. Two-way ANOVA, ^#^*p* = 0.0594, *p = 0.0225, **p = 0.0026. **D** Mice performed a novel object recognition task in which they were allowed to freely explore a box with two identical objects. Two hours later, they were exposed to one familiar object and one novel object, and time spent exploring each object was measured. Discrimination index: ((Time spent with novel object − Time spent with familiar object)/(Total time spent with both objects)). *N* = 7–10 mice per group. Error bars represent mean ± SEM

The delivery of Gas6 to the central nervous system to assess its utility as an immunomodulator has thus far been via intranasal administration or osmotic minipumps [21, 30]. We elected to test the hypothesis that chronic overexpression of Gas6 alleviates inflammation and plaque burden in aged APP/PS1 mice using an adeno-associated viral vector due to its long-term, stable gene expression without causing associated inflammation [31]. An adeno-associated virus containing the Gas6 construct under the *SYN1* promoter (AAV-Gas6) as well as a control virus that lacks the Gla and EGF domains of the Gas6 protein (AAV-control) and thus does not activate TAM receptors were produced [23] (Fig. 1A). To validate the efficacy of the viruses, AAV-Gas6 or AAV-control were injected into the hippocampi of 12-week-old male wild-type C57BL/6J mice. Immunohistochemistry for the DDK tag revealed distribution of viral transduction throughout the hippocampus (Additional file 1: Fig. S1A), with the higher dilution achieving better spread throughout the hippocampus. Western blot on hippocampal lysates revealed increased phospho-Axl in AAV-Gas6 hippocampi but not AAV-control hippocampi, indicating that Gas6 overexpression results in activation of Axl (Additional file 1: Fig. S1B, C). The higher AAV dosing was chosen due to greater spread of virus as visualized by DDK staining.

Aged APP/PS1 mice demonstrate deficits in a number of hippocampal dependent tasks, including those that assess recognition and conditioned memory such as the novel object recognition task and the conditioned and cued fear conditioning task [32]. To determine whether overexpression of Gas6 improves these behaviors, 9-month-old male and female APP/PS1 mice and their wild type littermates were injected with either AAV-Gas6 or AAV-control into bilateral hippocampi via stereotactic injection. Four weeks following injections, a battery of behavioral tasks including open field, novel object recognition, and contextual and cued fear conditioning were performed (Fig. 1B). In the open field task, mice were allowed to freely explore an open chamber and distance travelled and time spent in the center of the chamber were automatically quantified. Two-way ANOVA revealed a main effect of genotype on distance travelled, with APP/PS1 mice travelling more distance than their nontransgenic littermates [*F*_females_(1,33) = 10.62, *p* = 0.0026; *F*_males_(1,28) = 5.833, *p* = 0.0225]. There was no main effect of Gas6 overexpression on distance travelled or time spent in the center of the open field (Fig. 1C).

Mice then performed a novel object recognition task in which they were allowed to freely explore a box containing two identical objects for 5 min. Two hours later, mice were placed back in the box with one familiar object and one novel object. Nontransgenic mice did not perform significantly better than APP/PS1 mice and Gas6-treated groups did not differ from control-treated groups (Fig. 1D).

### Gas6 overexpression worsens contextual fear conditioning performance in male APP/PS1 mice

**Fig. 2:**
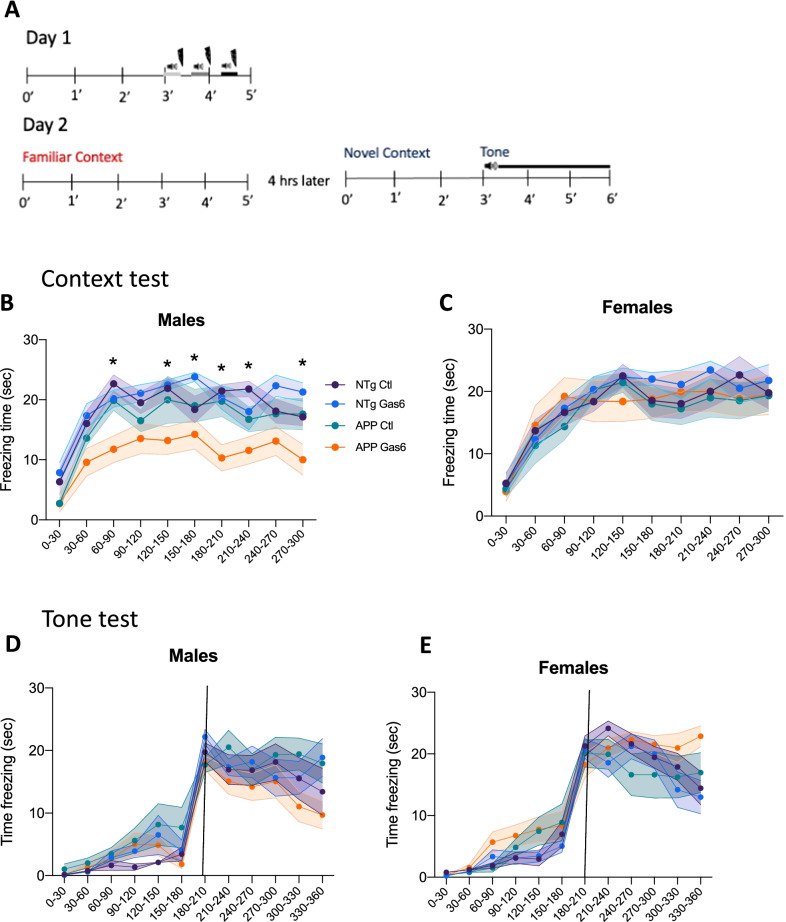
Gas6 overexpression worsens contextual fear conditioning performance in male APP/PS1 mice. **A** Schematic of contextual and cued fear conditioning task. **B**, **C** Freezing responses in each 30 s interval for male and female mice. N = 7–10 mice per group. Shaded areas represent mean ± SEM. Two-way ANOVA with Tukey’s multiple comparisons, *indicates APP-Gas6 group significantly differs from APP-control group for indicated 30 s time frame (*p* < 0.05). **D**, **E** Freezing responses in each 30 s interval for male and female mice during cued fear conditioning task in novel context. Vertical line indicates onset of conditioned tone. *N* = 7–10 mice per group. Upper and lower borders of shaded areas represent mean ± SEM

We assessed the effect of Gas6 overexpression on hippocampal-dependent and hippocampal-independent memory using a contextual and cued fear conditioning task. AAV-Gas6 and AAV-control treated mice were placed in a chamber in which they received three shock-tone pairings. Twenty-four hours later, mice were re-exposed to the familiar context and freezing responses were measured. Four hours following the context test, mice were exposed to a novel context for 3 min, after which the conditioned tone was played continuously for 3 min, and freezing responses were measured throughout (Fig. 2A). Freezing responses during the context test revealed a main effect of Gas6 treatment in male mice [*F*(7,61) = 2.803; *p* = 0.0135]. Post-hoc tests demonstrated that Gas6-treated male APP/PS1 mice froze less in the familiar context than control-treated APP/PS1 male mice or their nontransgenic littermates [*F*(1,271) = 6.975; *p* = 0.0087] (Fig. 2B). No effect of genotype or Gas6 treatment was found in female mice in the contextual task (Fig. 2C). For the cued fear conditioning task, there were no main effects of genotype or Gas6 treatment in male or female mice during the entire test period, nor at any individual time interval (Fig. 2D, E).

### Gas6 overexpression reduces plaque number in male APP/PS1 mice

**Fig. 3:**
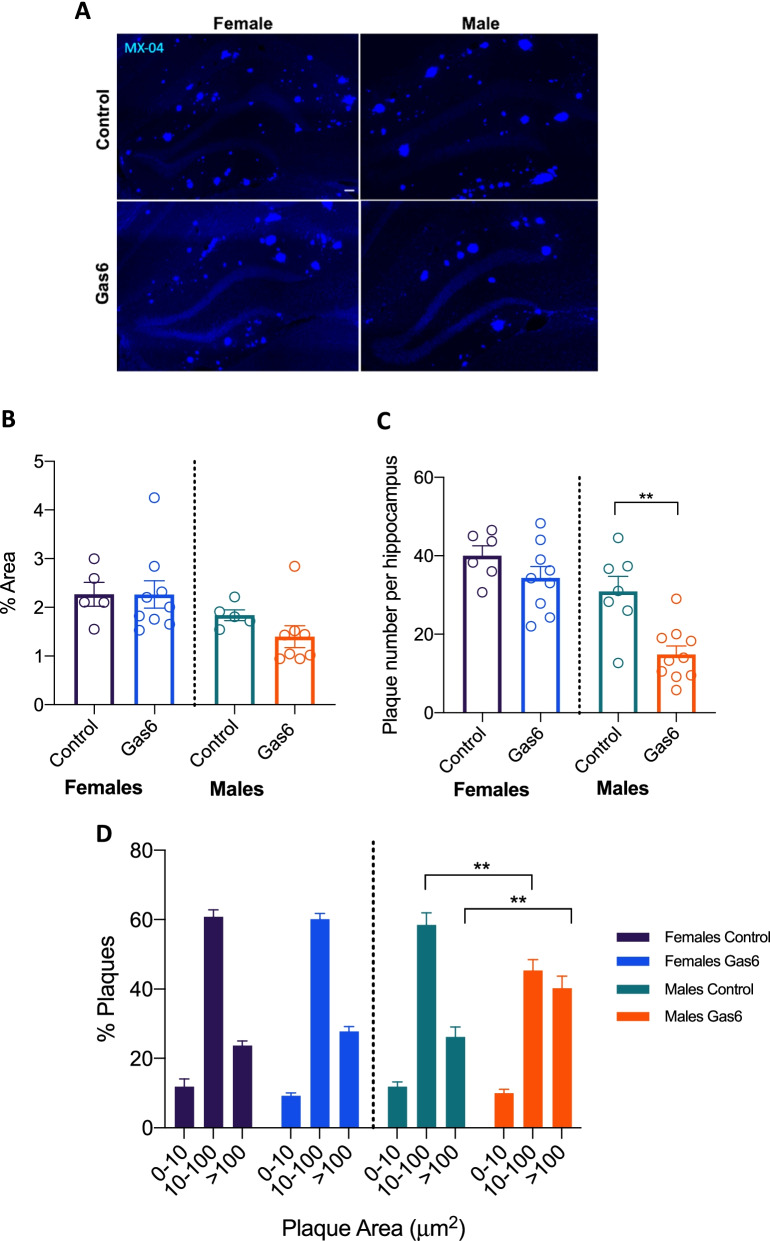
Gas6 expression reduces plaque number in male APP/PS1 mice. **A** Representative images from male and female APP mice injected with AAV-Gas6 or AAV-control at 9 months of age and injected with MX-04 24 h before sacrifice at 10 mo. Scale bar is 100 μm. **B**, **C** Quantitative measurements of % area of MX-04 and plaque numbers. *N* = 7–10 mice per group. Error bars represent mean ± SEM. Unpaired two-tailed *t*-test **p = 0.0014. **D** Particle analysis on MX-04 positive plaques. *N* = 7–10 mice per group. Error bars represent mean ± SEM. Two-way ANOVA **p < 0.01

Following behavioral testing, mice received an i.p. injection of methoxy-04 (MX-04), which binds amyloid plaques in vivo, and were sacrificed 24 h later. Analysis of MX-04 revealed no difference in MX-04-positive percent area (*p*_females_ = 0.9929; *p*_males_ = 0.1694) but a significant reduction in plaque number per hippocampus with Gas6 treatment in male mice [*t*(15) = 3.909; *p* = 0.0014] (Fig. 3A–C). No differences in percent area or plaque number were seen in female mice (*p*_%area_ = 0.9929; *p*_plaque number_ = 0.1905). To explore plaque size and number changes further, particle analysis was done and two-way ANOVA revealed an interaction between treatment and plaque size in male mice, with post-hoc tests demonstrating that Gas6-treated animals had significantly higher proportions of large plaques (> 100 µm^2^) and significantly lower proportions of medium size plaques (10–100 µm^2^) than control-treated male mice [*F*_interaction_(2,45) = 11.73, *p* < 0.0001; Control vs Gas6_large plaques,>100_ *p* = 0.0030; Control vs Gas6_Medium plaques 10–100_ *p* = 0.0058] (Fig. 3D).

### Evaluation of microglial activation markers following Gas6 overexpression

**Fig. 4:**
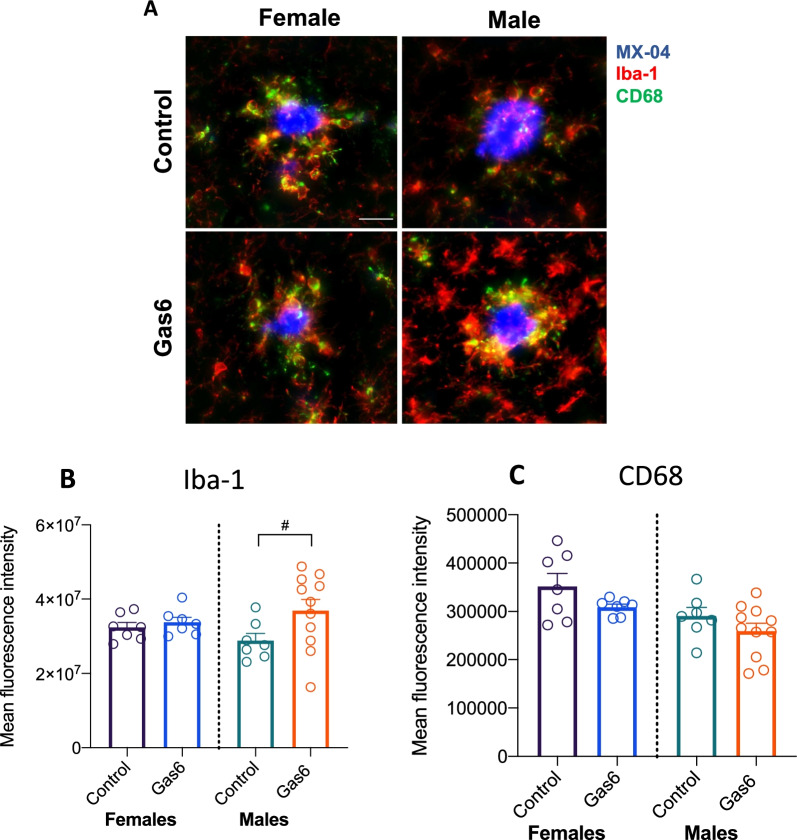
Effect of Gas6 on CD68 and Iba-1 expression in APP/PS1 mice. **A** Representative images and quantification of CD68 (**B**) and Iba-1 (**C**) staining on tissue from AAV-Gas6 or AAV-control mice. Scale bar represents 20 μm. *N* = 7–11 per group, 5–6 plaques per animal. Error bars represent mean ± SEM. Unpaired two-tailed *t*-test ^#^*p* = 0.0693

Treatment of peripheral dendritic cells and microglia with Gas6 in vitro reduces proinflammatory signaling [9, 33]. Thus, we hypothesized that overexpression of Gas6 would induce an anti-inflammatory microglial phenotype. Microglial activation was evaluated by immunohistochemistry on tissue from Gas6-treated and control-treated APP/PS1 mice. We found a trend toward increased Iba-1 fluorescence intensity around plaques in Gas6-treated compared to control-treated male mice (*t*(16) = 0.1.947, *p* = 0.0693) (Fig. 4A, B) but not in female mice (*t*(12) = 0.7414, *p* = 0.4725). There was no change in CD68 intensity in the area directly around plaques (*p*_females_ = 0.1414; *p*_males_ = 0.2258) (Fig. 4C).

### Gas6 does not alter microglial phagocytosis of amyloid beta in vitro or in vivo

**Fig. 5:**
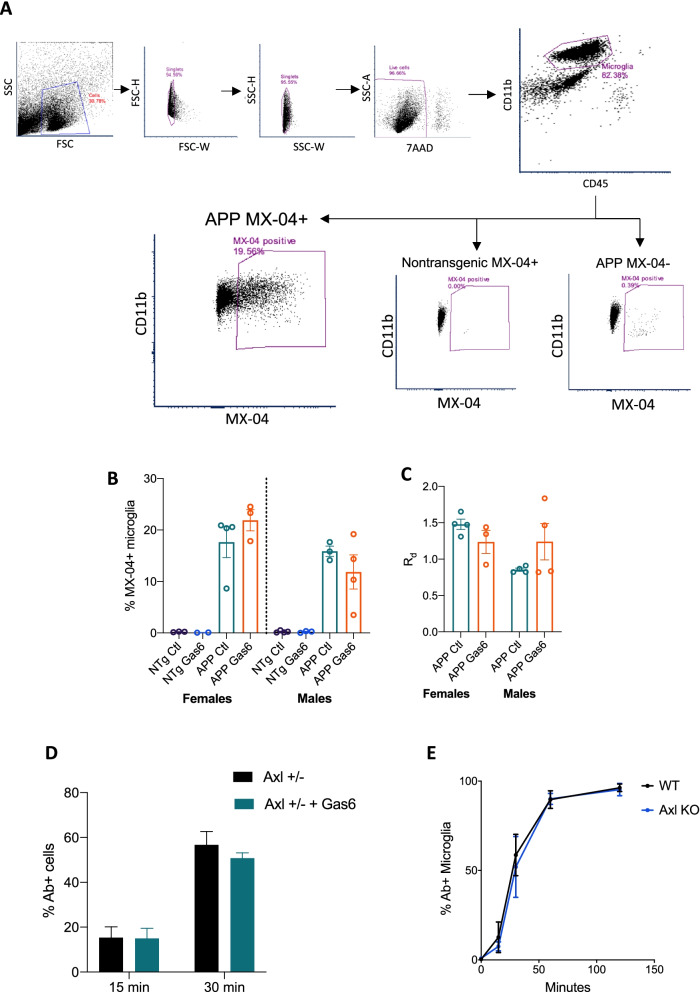
Gas6 does not affect microglial phagocytosis of amyloid beta in vivo or in vitro. **A** Ten-month-old APP/PS1 and nontransgenic littermates injected with AAV-Gas6 or AAV-control at 9 months of age were intraperitoneally injected with MX-04 24 h prior to sacrifice. Brains were processed and microglia (CD45*lo*/CD11b+) were analyzed by flow cytometry. Representative plots of gating strategy. **B, C** MX-04 + microglial populations showing percent of MX-04+ cells and relative median fluorescence per cell. *N* = 2–4 per group. Error bars represent mean ± SEM. **D** Primary microglia from Axl^+/−^ mice were treated with rmGas6 (50 nM) and beta-amyloid (1–42) HiLyte Fluor 488 for 15 or 30 min and analyzed by flow cytometry. *N* = 4–5 biological replicates per group. Error bars represent mean ± SEM. **E** Primary microglia from Axl + / − or Axl−/− P0-P2 pups were treated with rmGas6 for 15, 30, 60, or 120 min and analyzed by flow cytometry. *N* = 4–6 biological replicates per group. Error bars represent mean ± SEM

Activation of the Axl receptor on microglia by Gas6 is known to engage phagocytic machinery via Vav phosphorylation and Rac activation [8]. To assess whether overexpression of Gas6 in vivo stimulates microglial phagocytosis of amyloid beta plaques, MX-04 was injected 24 h before sacrifice, brains were processed for flow cytometry and microglia (CD45^lo^/CD11b^+^) were sorted (Fig. 5A). Approximately 18–20% of microglia were MX-04 positive, with no significant changes detected in percent of positive cells or relative median fluorescence intensity between Gas6 and control conditions (Fig. 5B, C). To assess whether Gas6 induces phagocytosis of fibrillar Aβ (fAβ), primary microglia were cultured with fluorescent fibrillar Aβ-42 with or without recombinant mouse Gas6. Addition of Gas6 did not increase the percent of fAβ positive microglia at 15 or 30 min (Fig. 5D). Furthermore, Axl knockout primary microglia did not demonstrate impairments in amyloid beta internalization (Fig. 5E).

### Gas6 overexpression induces a proinflammatory phenotype in microglia

**Fig. 6:**
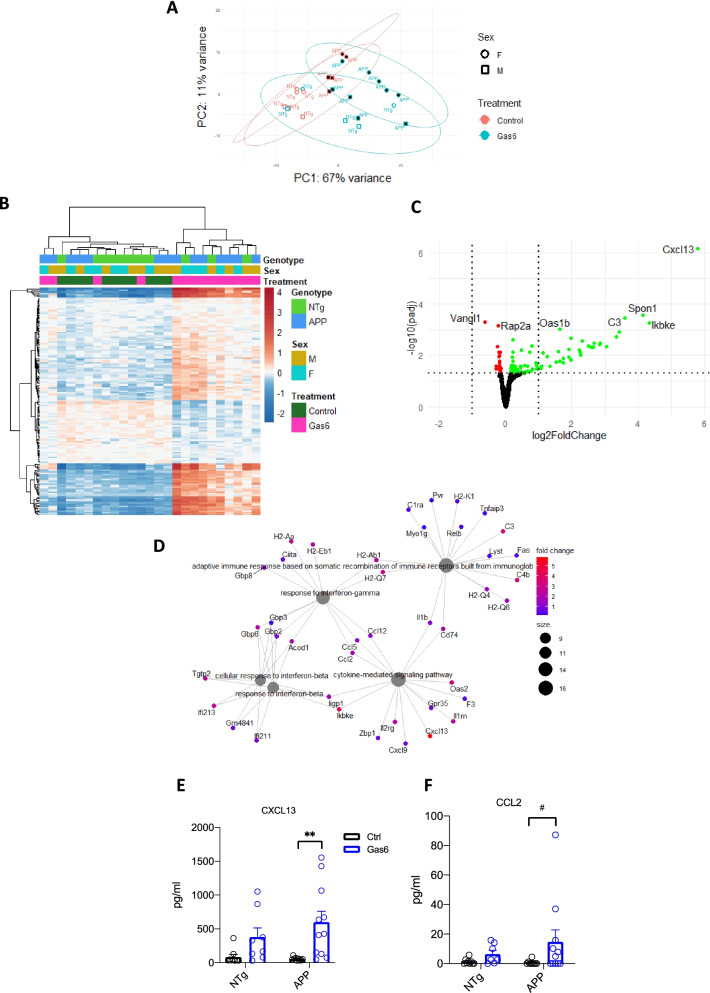
RNAseq of AAV-Gas6 treated APP/PS1 mice microglia reveals an enrichment of transcriptional signatures related to interferon response. **A** PCA plot of microglial samples. **B** Heatmap showing significantly DEGs between microglia isolated from AAV-Gas6 treated APP/PS1 mice and AAV-control hippocampi. Colors are shown in logarithmic scale. **C** Volcano plot with significantly downregulated genes (shown in red) and significantly upregulated genes (shown in green). Genes with a *p*-value < 0.001 are labeled. Vertical line is the *p* = 0.05 cutoff and horizontal lines show log2FoldChange = − 1 or 1. **D** Overrepresentation test of significantly upregulated genes through clusterProfiler package shows enrichment for interferon-gamma and beta response related pathways. **E**, **F** CXCL13 and CCL2 ELISA on hippocampal protein lysates from Gas6 and control-treated mice. Two-way ANOVA followed by Tukey post-hoc **p = 0.0018, ^#^*p* = 0.0878

To assess microglial transcriptional changes associated with Gas6 overexpression, microglia from Gas6-treated animals and control-treated animals were sorted and bulk RNAseq was performed. There were 98 upregulated and 41 downregulated differentially expressed genes (DEGs) between Gas6-treated APP/PS1 mice and control-treated APP/PS1 mice (Fig. 6A–C). Interestingly, overrepresentation test performed by clusterProfiler package on DEGs that were upregulated with Gas6 treatment demonstrated enrichment of interferon-gamma and beta response related pathways (Fig. 6D). ELISA analysis from hippocampal protein lysates on DEGs CXCL13 and CCL2 were completed to verify RNAseq findings; two-way ANOVA revealed a significant effect of Gas6 treatment on CXCL13 expression (*F*(1,34) = 14.02; *p* = 0.0007) with Tukey’s multiple comparisons test revealing a significant difference between Gas6-treated APP mice and control-treated APP mice (*p* = 0.0018), while a trend toward increased CCL2 expression with Gas6-treatment was found (*F*(1,32) = 3.608; *p* = 0.0666) (Fig. 6E, F).

## Discussion

Chronic neuroinflammation is a critical mediator of pathology in Alzheimer’s disease. Production of inflammatory cytokines early in the disease course may activate microglia and engage them to surround and engulf amyloid plaques in a manner that may be protective [34]. However, ongoing microglial activation and production of proinflammatory cytokines can damage surrounding neural parenchyma and lead to synapse loss [4, 5]. Because the addition of Gas6 is neuroprotective in a number of disease models [20, 21, 30]) and is pro-phagocytic, we hypothesized that Gas6 would improve behavior when overexpressed in aged APP/PS1 mice. In contrast, we found an interesting triad of worsened behavior, reduced plaque burden, and increased pro-inflammatory signaling, which is reminiscent of previous work from our lab in which chronic overexpression of IL-1β worsens behavior and improves plaque clearance in APP/PS1 mice. [24, 35, 36].

Gas6-treated APP/PS1 male mice froze less than control-treated APP/PS1 mice during the contextual fear conditioning task, which indicates a worsening of hippocampal-dependent memory, and were no different than AAV-control treated animals or wild type animals in the tone test, a measure of hippocampal-independent memory (Fig. 2). The mechanism by which overexpression of Gas6 worsens performance in the CFC task in a sex-specific manner is unknown, and is similar to recent evidence demonstrating a similar worsening of behavior in the fear conditioning task in the presumably opposite condition of APP/PS1 Axl^−/−^/Mertk^−/−^ mice [15]. One important caveat to highlight concerning the behavioral data is that there was no effect of genotype found in many of the performed behavioral tasks; nontransgenic and APP/PS1 mice performed similarly in the novel object recognition task and in the contextual and cued fear conditioning task. While previously published studies indicate behavioral deficits in the APP/PS1 model at this age, behavioral paradigms are highly variable between labs and many factors, including housing, animal diets, experimental temperatures, types of audio cues, animal handling differences, and differences in light/dark cycles, may all affect reproducibility of behavioral measures. Future experiments will seek to use alternative behavioral tasks or endpoints that elicit genotype differences.

Our data suggest that overexpression of Gas6 reduces plaque number in male mice but shifts the plaque size distribution towards larger, presumably dense-core, plaques. This agrees well with recent findings by Huang et al. who show that APP/PS1 Axl^−/−^Mertk^−/−^ mice have fewer dense-core plaques, with the overarching hypothesis that Gas6-TAM signaling promotes phagocytosis and thus exocytosis of amyloid which contributes to the formation of dense-core plaques [15]. Our data describe only methoxy-04-labeled amyloid beta and do not address changes in oligomeric forms of amyloid beta or production of amyloid beta peptide; future studies may seek to determine whether overexpression of Gas6 affects production of amyloid beta and whether oligomeric forms of amyloid beta are affected.

We did not find robust signs of increased phagocytosis in Gas6-treated APP/PS1 mice; Gas6 did not increase the percentage of MX-04+ microglia, the phagocytic marker CD68 was not differentially expressed between Gas6- and control-treated animals, and the addition of Gas6 did not increase the ability of primary microglia to phagocytose fibrillar amyloid beta in vitro. It is likely that additional Gas6 in our experiments has little effect on microglial phagocytosis given recent evidence that Gas6 is ubiquitous surrounding amyloid plaques and serves as a bridge between plaques and microglia [15]. Furthermore, it is possible that our low sample number for the MX-04+ phagocytosis experiment limits our ability to detect a change, and the primary microglia used in our in vitro experiments were unstimulated and thus may have expressed low levels of the Axl receptor at baseline, caveats that should be addressed in future experiments.

Bulk RNAseq of microglia revealed a striking upregulation of genes involved in IFN-β and IFN-γ mediated signaling in Gas6 treated vs control treated hippocampi, with upregulation of CCL2 and CX3CL13 confirmed by ELISA. CCL2 and its receptor CCR2 are known to increase microglial accumulation around amyloid plaques, and mice deficient in CCR2 have increased amyloid beta deposition [37]. CXCL13 is not expressed in the healthy CNS, but is upregulated in autoimmune and other neuroinflammatory environments [38, 39]. Thus, Gas6 seems to induce a proinflammatory environment that may improve microglial recruitment to plaques and contribute to the reduction in plaque number seen in the AAV-Gas6 treated mice. Axl is known to physically associate with and initiate signaling through the type I IFN-α receptor, followed by upregulation of anti-inflammatory genes *SOCS1* and *SOCS3* via STAT1 [9]. Although a direct link between Gas6 and type II interferon signaling has not been reported in the literature, complex crosstalk mediated by STAT1 that involves promotion of IFN-γ signaling by IFN-α/β occurs [40–42]. One potential mechanism to explore in future studies may be secondary upregulation of IFN-γ related genes following Gas6-Axl activation of IFN-α/β signaling. Our observation of upregulated pro-inflammatory signaling, and lack of differential expression of canonical Gas6-mediated anti-inflammatory responses conflicts with previous research describing an anti-inflammatory role of Gas6 both in vitro and in vivo [21, 43]. Together, these findings suggest an opposite, pro-inflammatory effect of Gas6 and prompt careful evaluation of the mechanism by which Gas6 is acting in the diseased brain. Although surprising, induction of a pro-inflammatory environment by Gas6 may be the link between Gas6 overexpression and worsened behavior, as it is well established that inflammatory environments contribute to synaptic loss. Future studies will seek to investigate synaptic changes following Gas6 overexpression.

One overarching caveat lies in the use of the control AAV vector, which contains the signal sequence at the N-terminus of the protein and the two LG domains, but lacks the Gla and EGF domains that are necessary for achieving TAM activation [23]. Thus, the control vector is able to bind Axl, which may inhibit the Axl receptor rather than acting as an inactive control. Future studies will seek to explore whether this attenuated Gas6 protein acts as an Axl inhibitor, as this discovery may be useful in further investigations of Axl function.

Although we focus our discussion on Axl, it is possible that overexpression of Gas6 activates the other TAM receptors Tyro3 or MerTK. Recent data suggests that MerTK plays a more significant role in the context of AD, as single mutant APP/PS1 Mertk^−/−^ mice had similar plaque reductions as APP/PS1 Axl^−/−^Mertk^−/−^ mice, while single mutant APP/PS1 Axl^−/−^ mice were more similar to APP/PS1 mice [15]. Tyro3 is expressed on neurons in the cerebral cortex and hippocampus, and its overexpression has an anti-amyloidogenic effect in vitro via reduction in pathologic forms of amyloid beta and BACE1 protein levels [44]. The same study demonstrated that 5xfAD mice heterozygous for *Tyro3* have increased plaque formation. However, the authors demonstrate that reductions in amyloid beta processing seen with *Tyro3* overexpression in vitro were inhibited by Gas6 in a concentration-dependent manner, which contradicts our finding that Gas6 reduced plaque number and suggests that Gas6-Tyro3 signaling may not be a significant event in vivo. Future studies should seek to determine the primary TAM receptor through which Gas6 exerts an effect in AD. Furthermore, TAM receptors are expressed on other glial cells, including astrocytes. Future studies should aim to address whether overexpression of Gas6 induces astrocytic changes that may contribute to plaque changes.

Finally, the sex-dependent effect of Gas6 is worth noting. Recent work has uncovered sex-specific expression of microglial genes, whereby microglia from male mice exhibit an elevated inflammatory signature, whereas microglia from female mice are neuroprotective [45]. Furthermore, previous work from our lab demonstrates that microglia from male mice have heightened expression of inflammatory markers CD68 and CD11b following cranial irradiation, which was not observed in female mice [46]. Differences in TAM receptor expression in wild type and murine models of Alzheimer’s disease have not been reported, but the possibility remains that Axl expression or activation is reduced in female mice, which may explain our sex-specific findings. The possibility that Gas6 has a more robust effect on male microglia should be explored in the future.

## Conclusions

We demonstrate a sex-dependent, beneficial role of Gas6 on plaque pathology. However, this is accompanied by increased proinflammatory signatures, suggesting an unexpected proinflammatory role for Gas6 in the CNS in the context of AD. This work utilizes a novel, chronic method of overexpression of Gas6 and provides evidence for the involvement of TAM receptor signaling in the pathogenesis of Alzheimer’s disease.

### Supplementary Information

**Additional file 1: Figure S 1.** A) Representative sections of DDK staining following AAV-Gas6 injection into the hippocampus of C57Bl/6J mice demonstrates spread of virus throughout the hippocampus. Scale bar = 100 μm. B, C) Western blot for pAxl and Axl on hippocampal lysates from mice treated with either 1E10 vg/mL or 1E12 vg/mL AAV-Gas6 or AAV-control. N=3-4 per group, error bars represent mean +/- SEM. Unpaired two-tailed t-test, *p=0.0107, ***p=0.0007.

## Data Availability

All data generated or analyzed during this study are available from the corresponding author on reasonable request. RNAseq datasets can be found at https://www.ncbi.nlm.nih.gov/geo/query/acc.cgi?acc=GSE171195.

## Abbreviations

AAV: Adeno-associated virus
AD: Alzheimer’s disease
CNS: Central nervous system
CFC: Contextual fear conditioning
DEG: Differentially expressed gene
fAβ: Fibrillar amyloid beta
MX-04: Methoxy-04
