# Correction: Gas6 induces infammation and reduces plaque burden but worsens behavior in a sex-dependent manner in the APP/PS1 model of Alzheimer’s disease

**DOI:** 10.1186/s12974-023-02926-3

**Published:** 2023-11-06

**Authors:** Laura D. Owlett, Berke Karaahmet, Linh Le, Elizabeth K. Belcher, Dawling Dionisio-Santos, John A. Olschowka, Michael R. Elliott, M. Kerry O’Banion

**Affiliations:** 1 Del Monte Institute for Neuroscience, Department of Neuroscience, University of Rochester, Rochester, NY USA; 2 Department of Microbiology, Immunology, and Cancer Biology, University of Virginia, Charlottesville, VA USA

**Correction** **:** **Journal of Neuroinfammation (2022) 19:38** **https://doi.org/10.1186/s12974-022-02397-y**

The part of the Data availability statement and Funding information section are missing in the original publication. The missing information are given below.

## Funding

Funding was provided by University of Rochester University Research Award: Targeting AXL in Alzheimer’s disease, Grant Nos. F30AG061939, NIH R01 AG030149, NIH R56 AG066397 from the National Institutes of Health, and the National Institute on Drug Abuse Intramural Research Program.

## Availability of data and materials

All data generated or analyzed during this study are available from the corresponding author on reasonable request. Viral vectors used in this work can be obtained from Addgene.org, plasmids 202,540 and 202,541. RNAseq datasets can be found at https://www.ncbi.nlm.nih.gov/geo/query/acc.cgi?acc=GSE171195.
