# Supplementary figures and images for: Gas6 induces inflammation and reduces plaque burden but worsens behavior in a sex-dependent manner in the APP/PS1 model of Alzheimer’s disease
Source: J Neuroinflammation. 2022 Feb 7;19:38. doi: 10.1186/s12974-022-02397-y (PMC8822838; doi:10.1186/s12974-022-02397-y)

## Slide 1
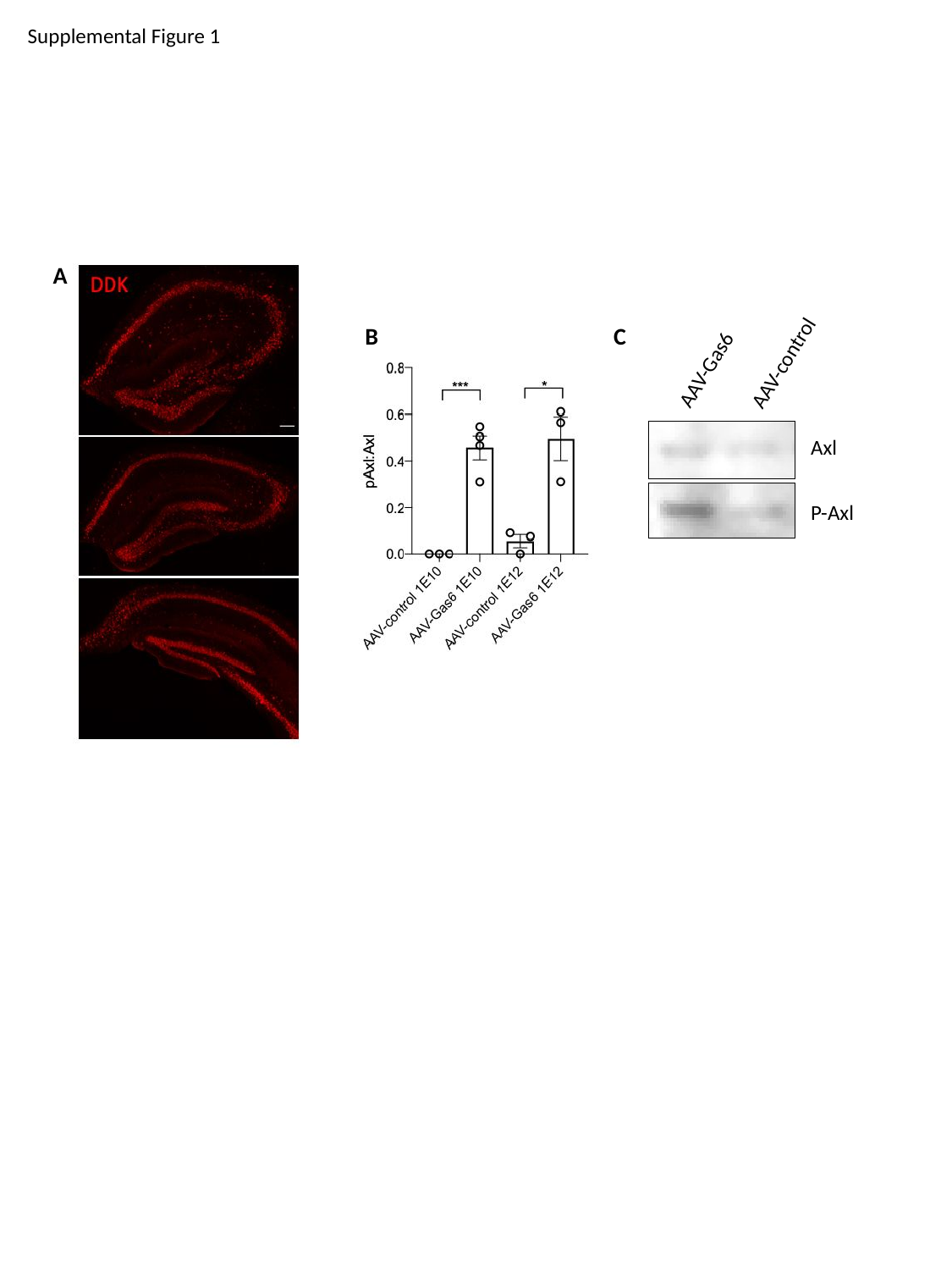

Supplemental Figure 1
A
B
C
AAV-control
AAV-Gas6
Axl
P-Axl

Supplement: Supplementary file 1 — Additional file 1: Figure S 1. A) Representative sections of DDK staining following AAV-Gas6 injection into the hippocampus of C57Bl/6J mice demonstrates spread of virus throughout the hippocampus. Scale bar = 100 μm. B, C) Western blot for pAxl and Axl on hippocampal lysates from mice treated with either 1E10 vg/mL or 1E12 vg/mL AAV-Gas6 or AAV-control. N=3-4 per group, error bars represent mean +/- SEM. Unpaired two-tailed t-test, *p=0.0107, ***p=0.0007. [file 12974_2022_2397_MOESM1_ESM.pptx]
